# Epidemiology of herpes simplex virus type 1 and genital herpes in Australia and New Zealand: systematic review, meta-analyses and meta-regressions

**DOI:** 10.1017/S0950268823000183

**Published:** 2023-02-08

**Authors:** Sawsan AlMukdad, Manale Harfouche, Uzma S. Farooqui, Lana Aldos, Laith J. Abu-Raddad

**Affiliations:** 1 Infectious Disease Epidemiology Group, Weill Cornell Medicine-Qatar, Cornell University, Qatar Foundation – Education City, Doha, Qatar; 2 World Health Organization Collaborating Centre for Disease Epidemiology Analytics on HIV/AIDS, Sexually Transmitted Infections, and Viral Hepatitis, Weill Cornell Medicine–Qatar, Cornell University, Qatar Foundation – Education City, Doha, Qatar; 3 Department of Population Health Sciences, Weill Cornell Medicine, Cornell University, New York, New York, USA; 4 Department of Public Health, College of Health Sciences, Member of QU Health, Qatar University, Doha, Qatar

**Keywords:** Australia, genital ulcer disease, herpes, meta-analysis, meta-regression, New Zealand, prevalence

## Abstract

Herpes simplex virus type 1 (HSV-1) infection is a lifelong infection that is acquired primarily orally and during childhood. We aimed to characterise HSV-1 epidemiology in Australia and New Zealand. HSV-1-related data as recent as 6 December 2021 were systematically reviewed, synthesised and reported, following PRISMA guidelines. Pooled mean seroprevalence and proportions of HSV-1 detection in genital ulcer disease (GUD) and in genital herpes were calculated using random-effects meta-analyses. Meta-regressions were also conducted. HSV-1 measures were retrieved from 21 eligible publications. Extracted HSV-1 measures included 13 overall seroprevalence measures (27 stratified) in Australia, four overall proportions of HSV-1 detection in clinically diagnosed GUD (four stratified) in Australia, and ten overall proportions of HSV-1 detection in laboratory-confirmed genital herpes (26 stratified) in Australia and New Zealand. Pooled mean seroprevalence among healthy adults in Australia was 84.8% (95% confidence interval (CI) 74.3–93.1%). Pooled mean seroprevalence was 70.2% (95% CI 47.4–88.7%) among individuals <35 years of age and 86.9% (95% CI 79.3–93.0%) among individuals ≥35 years. Seroprevalence increased by 1.05-fold (95% CI 1.01–1.10) per year. Pooled mean proportion of HSV-1 detection in GUD was 8.2% (95% CI 0.4–22.9%). Pooled mean proportion of HSV-1 detection in genital herpes was 30.5% (95% CI 23.3–38.3%), and was highest in young individuals. Proportion of HSV-1 detection in genital herpes increased by 1.04-fold (95% CI 1.00–1.08) per year. Included studies showed heterogeneity, but 30% of the heterogeneity in seroprevalence and 42% of the heterogeneity in proportion of HSV-1 detection in genital herpes were explained in terms of epidemiological factors. HSV-1 seroprevalence is higher in Australia than in other Western countries. HSV-1 epidemiology in Australia and New Zealand appears to be transitioning towards less oral acquisition in childhood, but more genital acquisition among youth.

## Introduction

Herpes simplex virus type 1 (HSV-1) infection is a lifelong infection that is acquired primarily orally and during childhood [1]. Although the infection is nearly always mild or asymptomatic, it can cause serious disease, including severe neurological, corneal and mucocutaneous complications [1, 2]. The historical pattern of HSV-1 epidemiology appears to be changing in Western countries, whereby HSV-1 oral acquisition is decreasing among children, but genital acquisition is increasing among youth, mainly due to oral sex [3–8]. To address HSV-1 disease burden and its evolving epidemiology, the World Health Organization (WHO) and other global partners are leading initiatives to better understand HSV-1 epidemiology and to develop a vaccine that prevents acquisition of infection [7, 9, 10].

HSV-1 epidemiology is well characterised in many Western countries [3, 5, 11, 12], but remains insufficiently understood in Australia, New Zealand and Pacific Island nations. Accordingly, we carried out a study to delineate trends and patterns to inform policy, programming and resource allocation, for the purpose of addressing the disease burden of this infection. The study implemented an established analytical approach that has been developed, tested and refined over years of investigation and applications for a range of infections [13–18].

We characterised HSV-1 epidemiology in this region by systematically reviewing available measures for HSV-1 antibody prevalence (seroprevalence), HSV-1 seroincidence, proportion of HSV-1 detection in clinically diagnosed genital ulcer disease (GUD), and proportion of HSV-1 detection in laboratory-confirmed genital herpes. We further estimated pooled means for HSV-1 seroprevalence and for proportions of HSV-1 detection in GUD and in genital herpes, and investigated associations and temporal trends for HSV-1 seroprevalence and for proportion of HSV-1 detection in genital herpes.

## Methods

The methodology used in this study was based on methodology we developed previously in a series of systematic reviews assessing HSV-1 epidemiology in other regions and countries [12, 15, 19–21]. A description of this methodology is shown in Box 1 and is outlined below.

Box 1. Description of study methodology.

**Table:** 

Methodology	Description
Data source and search strategy	- Search was conducted on 6 December, 2021 in PubMed and Embase. - Search strategies included exploded MeSH/Emtree terms and broad terms with no language or time restrictions. - The definition of Australia, New Zealand and Pacific Islands included 23 countries and territories: o Australia, New Zealand, American Samoa, Cook Islands, Fiji, French Polynesia, Guam, Kiribati, Marshall Islands, Micronesia, Nauru, New Caledonia, Niue, Northern Mariana Islands, Palau, Pitcairn, Samoa, Solomon Islands, Tokelau, Tonga, Tuvalu, Vanuatu, and Wallis and Futuna islands.
Study selection and inclusion and exclusion criteria	- Search results were imported into the reference manager Endnote (Thomson Reuters, USA). - Screening was performed in four stages: 1. Duplicate publications were identified and excluded. 2. Titles and abstracts were screened for relevant and potentially relevant publications. 3. Full texts of relevant and potentially relevant publications were retrieved and screened for relevance. 4. Bibliographies of relevant publications and reviews were checked for additional potentially relevant publications. - Inclusion criteria were any publication, with a minimum sample size of 10, reporting primary data on any of the following outcome measures: 1. HSV-1 antibody seroincidence as detected by a type-specific diagnostic assay. 2. HSV-1 seroprevalence as detected by a type-specific diagnostic assay. 3. Proportion of HSV-1 in GUD as detected by standard viral detection and subtyping methods. 4. Proportion of HSV-1 in genital herpes (as opposed to HSV-2) as detected by standard viral detection and subtyping methods. - Exclusion criteria were: o Case reports, case series, reviews, editorials, commentaries and qualitative studies. o Measures reporting seroprevalence in infants <6 months old as their antibodies can be maternal in origin. - In this study, the term ‘publication’ refers to a document reporting one or several outcome measures. ‘Study’ or ‘measure’ refers to a specific outcome measure and its details.
Data extraction and data synthesis	- Extracted variables included: author(s), publication title, year(s) of data collection, year of publication, country of origin, country of survey, city, study site, study design, study sampling procedure, study population and its characteristics (e.g., sex and age), sample size, HSV-1 outcome measures and diagnostic assay. - Stratification hierarchy for seroprevalence in descending order of preference was population type, age bracket (children versus adults) and age group: 1. Population type classified as: o Healthy general populations: healthy populations such as blood donors, pregnant women and outpatients with minor health conditions. o Clinical populations: any population with a major clinical condition or a condition related (potentially) to HSV-1 infection. o Other populations: other populations not satisfying above definitions or populations with an undetermined risk of acquiring HSV-1, such as HIV-positive patients, sex workers, men who have sex with men and prisoners. 2. Age bracket classified as: o Children: ≤15 years old individuals. o Adults: >15 years old individuals. 3. Age group classified as (groups optimised to best fit reported data): o <35 years old. o ≥35 years old. o Mixed age bands. - Stratification hierarchy for GUD and genital herpes included genital herpes episode status and study site: 1. Genital herpes episode status classified as: o Primary genital herpes. o Recurrent genital herpes. 2. Study site stratification classified as: o Hospital. o Sexually transmitted disease clinic.
Quality assessment	The Cochrane-informed approach for risk of bias assessment included: - Study's precision classification into low *versus* high based on the sample size (<100 *versus* ≥100). - Study's appraisal into low *versus* high risk of bias was determined using two quality domains: o Sampling method: probability-based *versu*s non-probability based. o Response rate: ≥80% *versus* <80% or unclear.
Meta-analyses	- Meta-analyses were conducted using DerSimonian-Laird random-effects models with inverse variance weighting. The variance of each outcome measure was stabilised using the Freeman-Tukey arcsine square-root transformation. - Pooled mean estimates for HSV-1 seroprevalence were estimated by population type, age bracket, age group, country, sex, year of publication category and year of data collection category. - Overall pooled mean proportion of HSV-1 detection in clinically diagnosed GUD cases was estimated. - Pooled mean proportion of HSV-1 detection in laboratory-confirmed genital herpes cases was estimated by age group, country, sex, year of publication category and year of data collection category. - Heterogeneity assessment was based on three complementary metrics: o Cochran's *Q* statistic to assess existence of heterogeneity in effect size (*P* value < 0.1 indicated heterogeneity). o *I*^2^ heterogeneity measure to assess the percentage of between-study variation in effect size that is due to actual differences in effect size rather than chance. o Prediction interval to describe the distribution of true outcome measures around the pooled mean.
Meta-regressions	- Univariable and multivariable random-effects meta-regression analyses using log-transformed proportions were carried out to identify predictors of HSV-1 seroprevalence and proportion of HSV-1 detection in laboratory-confirmed genital herpes. - Factors in the univariable model with a *P* value < 0.2 were included in the multivariable analysis. - Factors in the multivariable model with a *P* value ≤ 0.05 were deemed to be significant predictors. - Variables included in the univariable meta-regression model for HSV-1 seroprevalence were: o Age group. o Sex. o Population type. o Assay type (western blot and ELISA). o Sample size. o Sampling method. o Response rate. o Year of data collection. o Year of data collection category (≤2000; >2000). - Variables included in the univariable meta-regression model for proportion of HSV-1 detection in laboratory-confirmed genital herpes were: o Age group. o Sex. o Country. o Year of data collection. o Year of data collection category (≤2000; >2000).

Abbreviations: ELISA, enzyme-linked immunosorbent type-specific assay; GUD, genital ulcer disease; HSV-1, herpes simplex virus type 1; HSV-2, herpes simplex virus type 2.

### Data sources, search strategy, study selection and eligibility criteria

This systematic review was informed by the Cochrane Collaboration Handbook [22], and was reported according to the Preferred Reporting Items for Systematic Reviews and Meta-analyses (PRISMA) guidelines (Table S1) [23, 24]. The included Pacific Island nations were selected as informed by the United Nations regional geoscheme and the WHO definition of the Pacific region (Box 1) [25, 26]. Search strategies are shown in Table S2. Screening processes and inclusion and exclusion criteria are explained in Box 1.

Four outcomes were assessed: (1) HSV-1 seroincidence, whether defined as the occurrence of infection per person-time or as a cumulative risk over a specific duration, (2) HSV-1 seroprevalence defined as the proportion of individuals tested that were seropositive, (3) proportion of GUD cases in which HSV-1 was isolated as the cause of GUD and (4) proportion of genital herpes cases in which HSV-1 was isolated as the cause of the genital herpes.

### Data extraction, synthesis and quality assessment

Data extraction and double extraction were performed independently by four authors (SM, MH, UF and LA), using a predefined list of variables (Box 1). The validity of each HSV-1 diagnostic assay in each potentially relevant study was investigated in consideration of known limitations of HSV serology and potential cross reactivity between HSV-1 and HSV-2 antibodies [27, 28]. This was done with support of Professor Rhoda Ashley-Morrow of the University of Washington, an expert in HSV-1 diagnostic methods. Only studies that used valid and reliable assays were included. Each included study was subsequently appraised for precision and risk of bias (ROB), as informed by the Cochrane approach [22] and described in Box 1.

Both overall measures and stratified measures were extracted from relevant studies (Box 1). Since our aim was to characterise the natural heterogeneity that exists in HSV-1 epidemiology, measures were extracted and stratified by key epidemiological factors known to affect the natural epidemiology of this infection [12, 15, 19–21]. Meta-regression analyses were further conducted on these stratified measures to estimate effects of these epidemiological factors on both HSV-1 seroprevalence and proportion of HSV-1 detection in genital herpes.

### Meta-analyses

DerSimonian-Laird random-effects models [29] with the Freeman-Tukey double arcsine transformation [30] were used to conduct meta-analyses, after examining the transformation's applicability to the dataset [31]. Pooled mean estimates were calculated for HSV-1 seroprevalence and for proportions of HSV-1 detection in GUD and genital herpes. The meta package [32] was used to perform these analyses in R, version 4.0.4 (Box 1) [33].

Since our study approach implicitly treats each outcome for a specific stratum or subgroup as an independent measure, studies reporting outcomes for more strata or subgroups had larger weights in the pooled estimate than studies reporting outcomes for less strata or subgroups, even when the study sample size was comparable. In a sensitivity analysis, pooled estimates were calculated by first generating a single estimate for each study by pooling estimates of all strata and subgroups in that study, and then pooling the single estimates across all studies. Accordingly in this analysis studies had weights that are independent of the number of reported stratum or subgroup outcomes.

### Meta-regressions

Univariable and multivariable random-effects meta-regression analyses were conducted to investigate associations with HSV-1 seroprevalence and with proportion of HSV-1 detection in genital herpes, as well as to explain the between-study heterogeneity (Box 1). Random-effects meta-regression allows for residual heterogeneity (between-study heterogeneity not explained by the covariates) by assuming that the true effects follow a normal distribution around the linear predictor [34]. The metareg package [34, 35] was used to calculate log-transformed seroprevalence and log-transformed proportions and to investigate associations in Stata/SE version 16.1 (Box 1) [36].

## Results

### Search results and scope of evidence

**Fig. 1.**
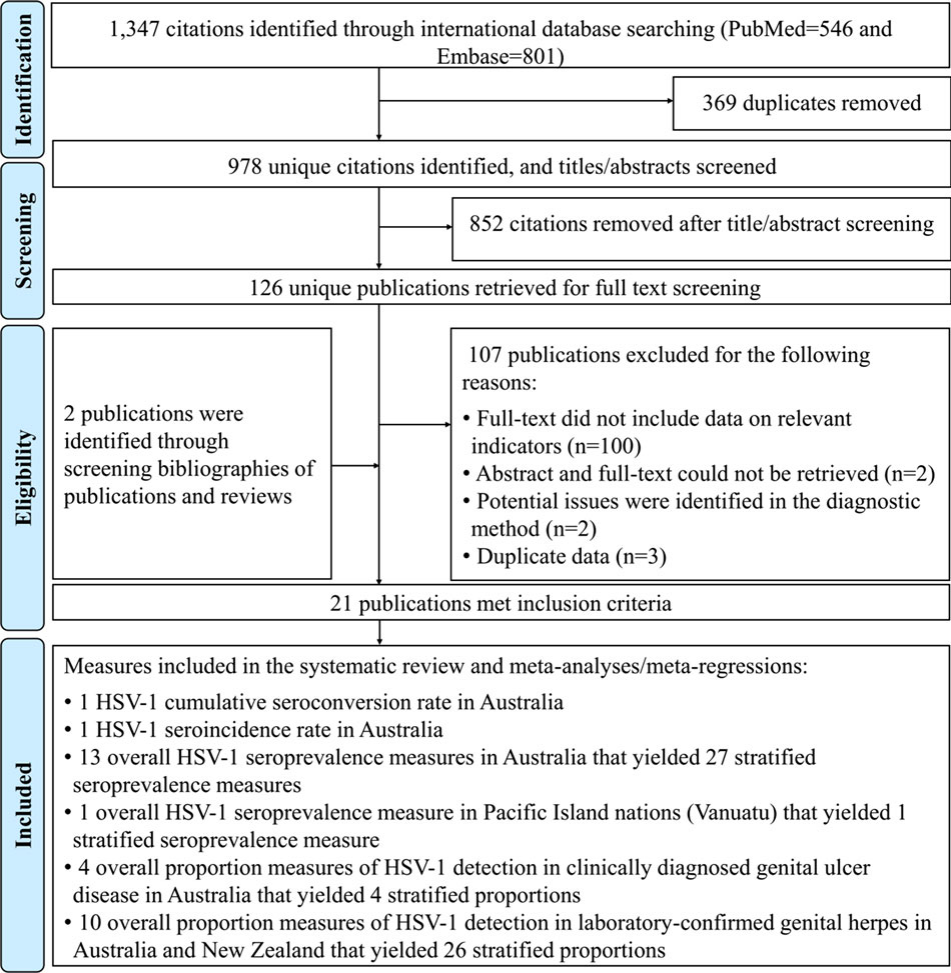
Flow chart of article selection for the systematic review of HSV-1 infection in Australia, New Zealand and Pacific Islands, per PRISMA guidelines [23]. Abbreviation: HSV-1, herpes simplex virus type 1.

Screening and study selection processes are described in Figure 1. The search retrieved 1347 records: 546 in PubMed and 801 in Embase, of which 19 were deemed relevant. Bibliographic screening identified two additional relevant studies [37, 38]. Altogether, 21 publications met the inclusion criteria from which data were extracted. Only one study was identified among the 21 Pacific Island nations, conducted in Vanuatu [39].

Extracted HSV-1 measures included one cumulative seroconversion rate and one seroincidence rate in Australia, 13 overall seroprevalence measures (27 stratified) in Australia, one overall seroprevalence measure in Vanuatu (one stratified), four overall proportions of HSV-1 detection in clinically diagnosed GUD (four stratified) in Australia, and ten overall proportions of HSV-1 detection in laboratory-confirmed genital herpes (26 stratified) in Australia and New Zealand. There were no seroprevalence studies in New Zealand and no GUD or genital herpes studies addressed any of the 21 Pacific Island nations.

### Cumulative seroconversion and seroincidence overview

One cohort study was identified that reported both cumulative seroconversion rate and seroincidence rate for HSV-1 infection among men who have sex with men (MSM) in Australia [40]. Cumulative seroconversion rate was estimated at 9.7% over a median follow-up duration of 2 years [40]. The seroincidence rate was estimated at 5.6 per 100 person-years [40].

### Seroprevalence overview

Overall HSV-1 seroprevalence measures are listed in Table S3. All extracted measures were from studies conducted in Australia (*n* = 13), apart from the one study conducted in Vanuatu [39]. Most studies were published prior to 2005 (number of measures (*n*) = 8; 61.5%) and used convenience sampling (*n* = 12; 92.3%).

Stratified HSV-1 seroprevalence measures in Australia for the different populations are summarised in Table 1. Seroprevalence across all populations (*n* = 27) ranged between 9.0–100.0% with a median of 75.5%. Seroprevalence among healthy general-population adults (*n* = 14) ranged between 32.5–100.0% with a median of 80.9%. Seroprevalence among clinical-population adults (*n* = 8) ranged between 9.0–73.5% with a median of 69.0%. Seroprevalence among MSM (*n* = 5) ranged between 54.2–87.7% with a median of 77.5%. No HSV-1 seroprevalence measures among children were identified in any of the countries.

**Table 1.** Pooled mean estimates for HSV-1 seroprevalence in Australia and Pacific Islands

Region/Populations	Outcome measures	Samples	HSV-1 seroprevalence		Pooled mean HSV-1 seroprevalence	Heterogeneity measures		
	Total *n*	Total *N*	Range	Median	Mean (95% CI)	*Q*^a^ (*P* value)	*I*^2^^b^ (%) (95% CI)	Prediction Interval^c^ (%)
**Australia**	**27**	**4898**	**9.0–100.0**	**75.5**	**76.4 (67.5–84.3)**	**1313.2 (*P* < 0.001)**	**98.0 (97.6–98.3)**	**24.9–100.0**
Healthy general populations								
Adults	14	2511	32.5–100.0	80.9	84.8 (74.3–93.1)	672.4 (*P* < 0.001)	98.1 (97.5–98.5)	33.3–100.0
Clinical populations								
Adults	8	1016	9.0–73.5	69.0	59.1 (41.2–75.9)	395.8 (*P* < 0.001)	98.2 (97.6–98.7)	4.3–100.0
Other populations								
Men who have sex with men	5	1371	54.2–87.7	77.5	75.7 (64.8–85.1)	39.2 (*P* < 0.001)	89.8 (79.0–95.0)	31.7–99.9
Sex								
Women	3	637	69.7–81.6	79.2	79.7 (76.5–82.8)	2.4 (*P* = 0.297)	17.5 (0.0–91.4)	56.1–95.9
Men	8	1971	9.0–87.7	74.5	66.6 (47.1–83.6)	598.4 (*P* < 0.001)	98.8 (98.5–99.1)	4.6–100.0
Mixed sexes	16	2290	32.5–100.0	76.1	80.8 (69.6–90.0)	659.3 (*P* < 0.001)	97.7 (97.1–98.2)	26.4–100.0
Age group								
<35 years	7	1650	32.5–100.0	67.0	70.2 (47.4–88.7)	403.4 (*P* < 0.001)	98.5 (97.9–98.9)	1.6–100.0
≥35 years	11	1724	75.1–100.0	81.5	86.9 (79.3–93.0)	87.9 (*P* < 0.001)	88.6 (81.6–93.0)	53.0–100.0
Mixed	9	1524	9.0–81.6	73.2	65.9 (49.5–80.6)	553.7 (*P* < 0.001)	98.6 (98.1–98.9)	9.9–100.0
Year of data collection category								
≤2000	15	2524	9.0–85.2	73.5	70.5 (60.5–79.6)	636.7 (*P* < 0.001)	97.8 (97.2–98.3)	26.6–98.8
>2000	12	2374	32.5–100.0	84.6	83.3 (68.3–94.3)	676.0 (*P* < 0.001)	98.4 (97.9–98.7)	18.0–100.0
Year of publication category								
≤2005	10	2161	9.0–81.6	71.4	62.5 (46.3–77.4)	715.8 (*P* < 0.001)	98.7 (98.4–99.0)	7.7–100.0
>2005	17	2737	44.8–100.0	80.1	83.5 (74.8–90.6)	250.3 (*P* < 0.001)	93.6 (91.2–95.4)	39.0–100.0
**Pacific Islands**	**1**^d^	**134**	–	–	**100.0** (**98.7**–**100.0)**	–	–	–
Healthy general populations								
Adults	1^d^	134	–	–	100.0 (98.7–100.0)	–	–	–
**All studies**	**28**	**5032**	**9.0**–**100.0**	**76**.**1**	**77.8** (**68.9**–**85.7)**	**1477.1 (*P* < 0.001)**	**98.2** (**97.8**–**98.5)**	**24.6**–**100.0**

Abbreviations: CI, confidence interval; HSV-1, herpes simplex virus type 1.

a *Q*: Cochran's *Q* statistic is a measure assessing heterogeneity in pooled outcome measures, here HSV-1 seroprevalence.

b *I*^2^: A measure assessing the magnitude of between-study variation due to true differences in HSV-1 seroprevalence across studies rather than to sampling variation.

c Prediction interval: A measure quantifying the 95% interval of the distribution of true HSV-1 seroprevalence around the estimated pooled mean.

d No meta-analysis was done due to the small number of studies (*n* < 3).

### Pooled mean estimates for HSV-1 seroprevalence

Pooled mean seroprevalence across all populations in Australia was 76.4% (95% confidence interval (CI) 67.5–84.3%; Table 1). Pooled means for seroprevalence among healthy adults and clinical adults were 84.8% (95% CI 74.3–93.1%) and 59.1% (95% CI 41.2–75.9%), respectively. Pooled mean seroprevalence for MSM was 75.7% (95% CI 64.8–85.1%). Pooled mean seroprevalence was 70.2% (95% CI 47.4–88.7%) among individuals <35 years of age and 86.9% (95% CI 79.3–93.0%) among individuals ≥35 years.

In the sensitivity analysis pooling estimates by first generating a single estimate for each study and then pooling the estimates across all studies, pooled mean seroprevalence across all populations in Australia was 66.3% (95% CI 48.5–81.9%). Pooled mean seroprevalence was 77.1% (95% CI 51.9–94.8%) among healthy adults, 52.2% (95% CI 26.3–77.5%) among clinical adults, and 75.7% (95% CI 73.4–78.0%) among MSM.

The majority of meta-analyses reflected heterogeneity (*P* value < 0.001) with wide prediction intervals (Table 1). The variation in seroprevalence resulted from true differences in seroprevalence as opposed to sampling variation (*I*^2^ > 50%). Forest plots of meta-analyses by population type are shown in Figure S1.

### Sources of between-study heterogeneity and predictors of HSV-1 seroprevalence

Univariable and multivariable meta-regression analyses for HSV-1 seroprevalence are shown in Table 2 and Table S4. Due to collinearity between year of data collection and year of publication, four multivariable models were conducted, two using a categorical variable for time and two using time as a continuous linear variable.

**Table 2.** Univariable and multivariable meta-regression analyses for HSV-1 seroprevalence in Australia^a^

	Outcome measures	Samples	Univariable analysis				Multivariable analysis^b^			
							Model 1^c^		Model 2^d^	
	Total *n*	Total *N*	*RR* (95% CI)	*P* value	LR test *P* value	Adjusted *R*^2^ (%)	*ARR* (95% CI)	*P* value	*ARR* (95% CI)	*P* value
*Population characteristics*										
Age group										
<35 years	7	1650	1.00	–	0.173	7.79	1.00	–	1.00	–
≥35 years	11	1724	1.35 (0.88–2.08)	0.162			1.35 (0.84–2.18)	0.197	1.40 (0.94–2.07)	0.092
Mixed	9	1524	0.95 (0.61–1.50)	0.832			1.61 (0.58–4.47)	0.344	2.45 (0.98–6.12)	0.054
Sex^e^										
Women	3	637	1.00	–	0.461	0.00	1.00	–	1.00	–
Men	8	1971	0.77 (0.41–1.45)	0.397			0.63 (0.25–1.58)	0.308	0.40 (0.17–0.97)	0.044
Mixed	16	2290	0.97 (0.54–1.75)	0.922			1.12 (0.45–2.79)	0.791	0.99 (0.45–2.18)	0.973
Population type										
Healthy general populations	14	2511	1.00	–	0.135	9.11	1.00	–	1.00	–
Clinical populations	8	1016	0.67 (0.45–1.00)	0.051			0.73 (0.34–1.56)	0.399	0.88 (0.45–1.70)	0.677
Other populations^f^	5	1371	0.94 (0.59–1.48)	0.779			1.60 (0.63–4.03)	0.304	2.39 (1.04–5.49)	0.040
*Study methodology characteristics*										
Assay type										
Western blot	17	3187	1.00	–	0.517	0.00	–	–	–	–
ELISA	10	1711	1.13 (0.78–1.64)	0.517			–	–	–	–
Sample size^g^										
<100	1	94	1.00	–	0.335	0.58	–	–	–	–
≥100	26	4802	1.59 (0.60–4.17)	0.335			–	–	–	–
Sampling method										
Probability based	6	1000	1.00	–	0.584	0.00	–	–	–	–
Non-probability based	21	3898	0.89 (0.58–1.37)	0.584			–	–	–	–
Response rate										
≥80%	5	1371	1.00	–	0.939	0.00	–	–	–	–
<80%	7	1637	0.91 (0.52–1.60)	0.734			–	–	–	–
Unclear	15	1890	0.93 (0.57–1.53)	0.772			–	–	–	–
*Temporal variables*										
Year of data collection category^e^										
≤2000	15	2524	1.00	–	0.478	0.00	1.00	–	–	–
>2000	12	2374	1.14 (0.79–1.63)	0.478			1.16 (0.68–1.99)	0.573	–	–
**Year of data collection**	27	4898	1.02 (0.99–1.05)	0.109	0.109	7.26	–	–	1.05 (1.01–1.10)	0.022

Abbreviations: *ARR*, adjusted risk ratio; CI, confidence interval; ELISA, enzyme-linked immunosorbent type-specific assay; HSV-1, herpes simplex virus type 1; *RR*, risk ratio.

a The only available study from the Pacific Islands nations (Vanuatu) was excluded from this analysis.

b Two multivariable models were conducted, one for year of data collection as a categorical variable and one for year of data collection as a linear term.

c Variance explained by the final multivariable model 1 (adjusted *R^2^*) = 4.96%.

d Variance explained by the final multivariable model 2 (adjusted *R^2^*) = 29.72%.

e Although sex and year of data collection category variables did not have a statistically significant association with the outcome in the univariable analysis (*P* value > 0.2), they were included in the multivariable analysis because of epidemiological relevance.

f Other populations included only men who have sex with men.

g Sample size denotes the sample size of each study population found in the original publication.

The model that included age group, sex, population type, and year of data collection as a continuous linear variable, explained 30% of seroprevalence variation (Table 2). Men had 0.40-fold (95% CI 0.17–0.97) lower seroprevalence than women. Seroprevalence increased by 1.05-fold (95% CI 1.01–1.10) per year. There was no evidence for differences in seroprevalence between healthy general populations and clinical populations, but there was some evidence of higher seroprevalence among MSM.

The other three multivariable models showed similar results, but evidence for the associations did not reach statistical significance (Table 2 and Table S4).

### HSV-1 detection in clinically diagnosed GUD and in laboratory-confirmed genital herpes

Overall proportions of HSV-1 detection in clinically diagnosed GUD and in laboratory-confirmed genital herpes are listed in Table S5 and their stratified measures are summarised in Table 3. Proportion of HSV-1 detection in GUD (*n* = 4) ranged from 1.1–30.0% with a median of 7.5% and a pooled mean of 8.2% (95% CI 0.4–22.9%).

**Table 3.** Pooled mean proportions of HSV-1 detection in clinically diagnosed genital ulcer disease and in laboratory-confirmed genital herpes in Australia and New Zealand

Population type	Outcome measures	Samples	Proportion of HSV-1 detection (%)		Pooled mean proportion of HSV-1 detection (%)	Heterogeneity measures		
	Total *n*	Total *N*	Range	Median	Mean (95% CI)	*Q*^a^ (*P* value)	*I*^2^^b^ (%) (95% CI)	Prediction Interval^c^ (%)
Patients with clinically diagnosed GUD								
**All patients with GUD**	**4**	**6478**	**1.1–30.0**	**7**.**5**	**8.2** (**0.4–22.9)**	**61.1 (*P* < 0.001)**	**95.1** (**90.4–97.5)**	**0.0–91.2**
*Patients with laboratory-confirmed genital herpes*								
Country								
Australia	23	34 937	5.6–80.0	36.0	32.5 (24.8–40.7)	2979.4 (*P* < 0.001)	99.3 (99.2–99.4)	2.8–74.2
New Zealand	3	3933	25.6–53.0	30.4	17.5 (3.3–39.5)	508.1 (*P* < 0.001)	99.6 (99.4–99.7)	0.0–100.0
Sex								
Women	9	15 421	19.0–80.0	38.8	40.7 (27.5–54.7)	672.3 (*P* < 0.001)	98.8 (98.4–99.1)	2.6–88.0
Men	9	10 606	5.6–60.5	25.0	24.5 (13.9–36.9)	204.7 (*P* < 0.001)	96.1 (94.2–97.4)	0.0–71.7
Mixed	8	12 843	10.1–53.0	33.8	26.6 (15.3–39.6)	1765.2 (*P* < 0.001)	99.6 (99.5–99.7)	0.0–76.4
Age group								
<35 years	8	5903	30.4–80.0	54.3	44.7 (26.3–63.9)	1548.1 (*P* < 0.001)	99.5 (99.4–99.6)	0.0–98.9
≥35 years	9	6288	5.6–38.8	22.0	19.2 (13.4–25.7)	171.7 (*P* < 0.001)	95.3 (93.0–96.9)	2.9–44.5
Mixed	9	26 679	10.1–49.7	36.0	31.0 (22.0–40.7)	1672.9 (*P* < 0.001)	99.5 (99.4–99.6)	4.4–67.5
Year of data collection category								
≤2000	12	29 765	10.1–55.5	24.6	26.0 (18.2–34.7)	1857.8 (*P* < 0.001)	99.4 (99.3–99.5)	2.3–63.2
>2000	14	9105	5.6–80.0	37.6	35.1 (23.2–48.0)	1710.5 (*P* < 0.001)	99.2 (99.1–99.4)	0.3–85.8
Year of publication category								
≤2005	10	27 913	10.1–55.5	22.1	24.3 (15.8–33.9)	1723.8 (*P* < 0.001)	99.5 (99.4–99.6)	1.0–64.0
>2005	16	10 957	5.6–80.0	37.6	35.1 (24.7–46.3)	1770.2 (*P* < 0.001)	99.2 (99.0–99.3)	1.4–82.1
**All patients with genital herpes**	**26**	**38 870**	**5.6–80.0**	**33**.**2**	**30.5** (**23.3–38.3)**	**3783.1 (*P* < 0.001)**	**99.3** (**99.3–99.4)**	**1.9–73.0**

Abbreviations: CI, confidence interval; GUD, genital ulcer disease; HSV-1, herpes simplex virus type 1.

a *Q*: Cochran's *Q* statistic is a measure assessing heterogeneity in pooled outcome measures, here proportions of HSV-1 detection.

b *I*^2^: A measure assessing the magnitude of between-study variation due to true differences in proportions of HSV-1 detection across studies rather than to sampling variation.

c Prediction interval: A measure quantifying the 95% interval of the distribution of true proportions of HSV-1 detection around the estimated pooled mean.

Proportion of HSV-1 detection in genital herpes (*n* = 26) ranged from 5.6–80.0% with a median of 33.2% and a pooled mean of 30.5% (95% CI 23.3–38.3%). In Australia (*n* = 23), the proportion ranged from 5.6–80.0% with a median of 36.0% and a pooled mean of 32.5% (95% CI 24.8–40.7%). In New Zealand (*n* = 3), it ranged from 25.6–53.0% with a median of 30.4% and a pooled mean of 17.5% (95% CI 3.3–39.5%). Among women (*n* = 9), the proportion ranged from 19.0–80.0% with a median of 38.8% and a pooled mean of 40.7% (95% CI 27.5–54.7%). Among men (*n* = 9), it ranged from 5.6–60.5% with a median of 25.0% and a pooled mean of 24.5% (95% CI 13.9–36.9%).

All meta-analyses showed evidence of heterogeneity (*P* value < 0.001) and wide prediction intervals (Table 3). Most of the heterogeneity was due to true differences in these proportions rather than sampling variation (*I*^2^ > 50%). Forest plots for meta-analyses of proportion of HSV-1 detection in GUD and proportion of HSV-1 detection in genital herpes are illustrated in Figure S2.

### Sources of between-study heterogeneity and predictors of HSV-1 detection in genital herpes

Results of univariable and multivariable meta-regressions for the proportion of HSV-1 detection in laboratory-confirmed genital herpes are shown in Table 4. The model including age group, sex, country and year of data collection as a continuous linear variable explained 42% of the variation in the proportion of HSV-1 detection. Individuals ≥35 years of age had 0.49-fold (95% CI 0.27–0.91) lower proportion of HSV-1 detection compared to individuals <35 years. Proportion of HSV-1 detection increased by 1.04-fold (95% CI 1.00–1.08) per year. The second model, including year of data collection as a categorical variable, showed similar overall results (Table 4).

**Table 4.** Univariable and multivariable meta-regression analyses for HSV-1 detection in laboratory-confirmed genital herpes in Australia and New Zealand

	Outcome measures	Samples	Univariable analysis				Multivariable analysis^a^			
							Model 1^b^		Model 2^c^	
	Total *n*	Total *N*	*RR* (95% CI)	*P* value	LR test *P* value	Adjusted *R*^2^ (%)	*ARR* (95% CI)	*P* value	*ARR* (95% CI)	*P* value
Age group										
<35	8	5903	1.00	–	0.165	6.54	1.00	–	1.00	–
≥35	9	6288	0.51 (0.25–1.05)	0.066			0.48 (0.25–0.92)	0.028	0.49 (0.27–0.91)	0.026
Mixed	9	26 679	0.80 (0.39–1.62)	0.523			0.68 (0.34–1.36)	0.256	0.69 (0.36–1.32)	0.245
Sex^d^										
Women	9	15 421	1.00	–	0.243	3.70	1.00	–	1.00	–
Men	9	10 606	0.60 (0.29–1.23)	0.153			0.63 (0.34–1.18)	0.141	0.63 (0.35–1.13)	0.116
Mixed	8	12 843	0.59 (0.29–1.22)	0.146			0.85 (0.37–1.94)	0.677	0.71 (0.33–1.50)	0.347
Country										
Australia	23	34 937	1.00	–	0.089	9.25	1.00	–	1.00	–
New Zealand	3	3933	0.47 (0.19–1.13)	0.089			0.30 (0.09–0.96)	0.043	0.40 (0.15–1.07)	0.065
Year of data collection category^d^										
≤2000	12	29 765	1.00	–	0.395	0.00	1.00	–	–	–
>2000	14	9105	1.28 (0.70–2.36)	0.395			1.61 (0.91–2.86)	0.094	–	–
Year of data collection	26	38 870	1.04 (0.99–1.09)	0.068	0.068	12.67	–	–	1.04 (1.00–1.08)	0.026

Abbreviations: *ARR*, adjusted risk ratio; CI, confidence interval; HSV-1, herpes simplex virus type 1; LR, likelihood ratio; *RR*, risk ratio.

a Two multivariable models were conducted, one for year of data collection as a categorical variable and one for year of data collection as a linear term.

b Variance explained by the final multivariable model 1 (adjusted *R^2^*) = 32.97%.

c Variance explained by the final multivariable model 2 (adjusted *R^2^*) = 41.54%.

d Although sex and year of data collection category variables did not have a statistically significant association with the outcome in the univariable analysis (p-value>0.2), they were included in the multivariable analysis because of epidemiological relevance.

### Quality assessment

Quality assessment results for the seroprevalence measures are presented in Table S6. Briefly, all studies (100.0%) had high precision. One study (7.1%) had low ROB in the sampling method domain, and one study (7.1%) had low ROB in the response rate domain. In contrast, 13 studies (92.9%) had high ROB in the sampling method domain, and two studies (14.3%) had high ROB in the response rate domain. None of the studies had low ROB in both quality domains, while one study (7.1%) had high ROB in both quality domains. The ROB assessment for the response rate domain was ‘unclear’ for 11 studies (78.6%).

## Discussion

About 80% of the Australian population is infected with HSV-1, a higher level than that observed in Western countries (74% in Europe, 58% in the United States of America (USA) and 51% in Canada) [3, 5, 12, 41], but comparable to levels in Asia (77%) [15] and Latin America and the Caribbean (LAC) (85%) [20], and lower than those in Africa (96%) [21] and the Middle East and North Africa (89%) [19]. HSV-1 seroprevalence also appears to be increasing, contrary to other global regions where seroprevalence is decreasing or has remained stable for the last three decades [3, 5, 12, 15, 19–21, 42], perhaps because of increasing immigration from regions with higher HSV-1 seroprevalence. The only other country in which evidence suggests increasing HSV-1 seroprevalence is Canada [41], another country of increasing immigration from regions with higher HSV-1 seroprevalence.

Seroprevalence was lower in men than women, an observation not seen elsewhere, except in Europe [12] and Canada [41], and contrary to the global pattern of no differences in seroprevalence by sex [15, 19–21]. While none of the included studies were on children <15 years of age, one study was conducted on senior high school students (age range of 15–20 years), and showed a low HSV-1 seroprevalence of 32.5% [43]. There was no evidence for differences in seroprevalence between healthy general populations and clinical populations, just as observed in other regions [12, 15, 19–21, 41], reflecting that this infection is a general population infection.

The proportion of HSV-1 (*versus* HSV-2) detection in genital herpes in Australia and New Zealand was relatively high at 31%, comparable to levels observed in other Western countries (34% in Europe, 33% in the USA and 37% in Canada) [12, 41, 44], but considerably higher than those in other regions (19% in Asia, 11% in LAC and 1% in Africa) [15, 20, 21]. The proportion of HSV-1 detection in genital herpes was also increasing year by year, as in other Western countries [3, 4, 8, 12, 41, 44]. These results are consistent with results of a study of HSV-2 infection in Australia and New Zealand that estimated HSV-2's contribution to genital herpes at 72% and decreasing year by year [45].

These findings suggest that HSV-1 epidemiology is transitioning in Australia and New Zealand towards less oral acquisition in childhood, but more genital acquisition among youth, as is occurring in other Western countries [3–8, 12, 41, 44]. This shift in epidemiology is associated with increasing adverse psychosexual effects among youth such as negative consequences for sexual relations and quality of life, depression, anxiety and low self-esteem [46–49]. This conclusion is supported by the higher proportion of HSV-1 detection in genital herpes among younger compared to older adults (Table 4). This conclusion also suggests that the level of HSV-1 seroprevalence among children in Australia and New Zealand is substantially lower than among adults, but no data among children were available to confirm this conjecture.

This study has limitations. There were hardly any data from Pacific Island nations; only one seroprevalence study was available in these countries. No seroprevalence studies were found in New Zealand. While there were multiple studies in Australia, none of them included children. Most seroprevalence studies were two-decades old, with no studies in recent years, where seroprevalence is more likely to be decreasing, especially among youth, as in other Western countries [3, 5, 12, 42]. Overall, the number of studies was not large enough to allow precise quantification of different potential associations through meta-regression analysis. Studies varied in methodological characteristics, including assay type, sample size, sampling method and response rate. Despite these limitations, the meta-regressions explained a substantial fraction of the heterogeneity observed across studies in terms of epidemiological factors such age (Tables 2 and 4 and Table S4).

## Conclusions

HSV-1 seroprevalence is high in Australia and higher than that in other Western countries. HSV-1 epidemiology in Australia and New Zealand may be transitioning towards less oral acquisition in childhood, but more genital acquisition among youth. HSV-1 infection is already the cause of nearly one-third of genital herpes cases, particularly among youth. There is an immediate need for expansion of HSV-1 epidemiological research in Pacific Island nations, in addition to Australia and New Zealand. This research should include studies on different populations with a focus on levels of infection in children. Research and surveillance are also needed to monitor the aetiology of GUD and genital herpes in this part of the globe.

## Data Availability

All relevant data are presented in the manuscript and its Supplementary Material file.

## Supplementary material

For supplementary material accompanying this paper visit http://doi.org/10.1017/S0950268823000183. click here to view supplementary material
