# Supplementary material for: Epidemiology of herpes simplex virus type 1 and genital herpes in Australia and New Zealand: systematic review, meta-analyses and meta-regressions
Source: Epidemiol Infect. 2023 Feb 8;151:e33. doi: 10.1017/S0950268823000183 (PMC9990408; doi:10.1017/S0950268823000183)
Supplement: Supplementary file 1 [file S0950268823000183sup001.docx]

**Supplementary Material**

**Table of Contents**

[**Table S1.** Preferred Reporting Items for Systematic Reviews and Meta-analyses (PRISMA) checklist [1]. 2](#_Toc112510904)

[**Table S2.** Data sources and search criteria for systematically reviewing HSV-1 epidemiology in Australia, New Zealand, and Pacific Islands. 5](#_Toc112510905)

[**Table S3.** Studies reporting HSV-1 seroprevalence among different populations in Australia and Pacific Island nations. 6](#_Toc112510906)

[**Figure S1.** Forest plots for the pooled mean HSV-1 seroprevalence among different populations in Australia. 7](#_Toc112510907)

[**Table S4**. Univariable and multivariable meta-regression analyses for HSV-1 seroprevalence in Australia^a^ using the year of publication instead of the year of data collection as the time variable. 8](#_Toc112510908)

[**Table S5.** Studies reporting proportions of HSV-1 detection in clinically diagnosed genital ulcer disease and in laboratory-confirmed genital herpes in Australia and New Zealand. 9](#_Toc112510909)

[**Figure S2.** Forest plots for the pooled mean proportions of HSV-1 detection in clinically diagnosed genital ulcer disease and in laboratory-confirmed genital herpes in Australia and New Zealand. 10](#_Toc112510910)

[1) Patients with clinically diagnosed genital ulcer disease. 10](#_Toc112510911)

[2) Patients with laboratory-confirmed genital herpes. 10](#_Toc112510912)

[**Table S6.** Summary of the precision assessment and risk of bias assessment for the studies reporting HSV-1 seroprevalence in Australia and Pacific Island nations. 11](#_Toc112510913)

[**References** 12](#_Toc112510914)

# **Table S1.** Preferred Reporting Items for Systematic Reviews and Meta-analyses (PRISMA) checklist [1].

| **Section and Topic** | **Item #** | **Checklist item** | **Location where item is reported** |
| --- | --- | --- | --- |
| **TITLE** | | |  |
| Title | 1 | Identify the report as a systematic review. | p. 1 |
| **ABSTRACT** | | |  |
| Abstract | 2 | See the PRISMA 2020 for Abstracts checklist. | p. 2 |
| **INTRODUCTION** | | |  |
| Rationale | 3 | Describe the rationale for the review in the context of existing knowledge. | p. 3 |
| Objectives | 4 | Provide an explicit statement of the objective(s) or question(s) the review addresses. | p .3 |
| **METHODS** | | |  |
| Eligibility criteria | 5 | Specify the inclusion and exclusion criteria for the review and how studies were grouped for the syntheses. | p. 4; Box 1 |
| Information sources | 6 | Specify all databases, registers, websites, organisations, reference lists and other sources searched or consulted to identify studies. Specify the date when each source was last searched or consulted. | Box 1 |
| Search strategy | 7 | Present the full search strategies for all databases, registers and websites, including any filters and limits used. | Table S2 |
| Selection process | 8 | Specify the methods used to decide whether a study met the inclusion criteria of the review, including how many reviewers screened each record and each report retrieved, whether they worked independently, and if applicable, details of automation tools used in the process. | p. 4; Box 1 |
| Data collection process | 9 | Specify the methods used to collect data from reports, including how many reviewers collected data from each report, whether they worked independently, any processes for obtaining or confirming data from study investigators, and if applicable, details of automation tools used in the process. | p. 4; Box 1 |
| Data items | 10a | List and define all outcomes for which data were sought. Specify whether all results that were compatible with each outcome domain in each study were sought (e.g. for all measures, time points, analyses), and if not, the methods used to decide which results to collect. | Box 1 |
|  | 10b | List and define all other variables for which data were sought (e.g. participant and intervention characteristics, funding sources). Describe any assumptions made about any missing or unclear information. | Box 1 |
| Study risk of bias assessment | 11 | Specify the methods used to assess risk of bias in the included studies, including details of the tool(s) used, how many reviewers assessed each study and whether they worked independently, and if applicable, details of automation tools used in the process. | p. 4-5; Box 1 |
| Effect measures | 12 | Specify for each outcome the effect measure(s) (e.g. risk ratio, mean difference) used in the synthesis or presentation of results. | p. 5; Box 1 |
| Synthesis methods | 13a | Describe the processes used to decide which studies were eligible for each synthesis (e.g. tabulating the study intervention characteristics and comparing against the planned groups for each synthesis (item #5)). | p. 4-5; Box 1 |
|  | 13b | Describe any methods required to prepare the data for presentation or synthesis, such as handling of missing summary statistics, or data conversions. | Box 1 |
|  | 13c | Describe any methods used to tabulate or visually display results of individual studies and syntheses. | Box 1 |
|  | 13d | Describe any methods used to synthesize results and provide a rationale for the choice(s). If meta-analysis was performed, describe the model(s), method(s) to identify the presence and extent of statistical heterogeneity, and software package(s) used. | p. 5; Box 1 |
|  | 13e | Describe any methods used to explore possible causes of heterogeneity among study results (e.g. subgroup analysis, meta-regression). | p. 6; Box 1 |
|  | 13f | Describe any sensitivity analyses conducted to assess robustness of the synthesized results. | p. 5; Box 1 |
| Reporting bias assessment | 14 | Describe any methods used to assess risk of bias due to missing results in a synthesis (arising from reporting biases). | NA |
| Certainty assessment | 15 | Describe any methods used to assess certainty (or confidence) in the body of evidence for an outcome. | NA |
| **RESULTS** | | |  |
| Study selection | 16a | Describe the results of the search and selection process, from the number of records identified in the search to the number of studies included in the review, ideally using a flow diagram. | p. 6; Figure 1 |
|  | 16b | Cite studies that might appear to meet the inclusion criteria, but which were excluded, and explain why they were excluded. | Figure 1 |
| Study characteristics | 17 | Cite each included study and present its characteristics. | p. 7; Tables S3 and S5 |
| Risk of bias in studies | 18 | Present assessments of risk of bias for each included study. | p. 10; Table S6 |
| Results of individual studies | 19 | For all outcomes, present, for each study: (a) summary statistics for each group (where appropriate) and (b) an effect estimate and its precision (e.g. confidence/credible interval), ideally using structured tables or plots. | p. 7; Tables 1 and 3; Figures S1 and S2; Tables S3 and S5 |
| Results of syntheses | 20a | For each synthesis, briefly summarise the characteristics and risk of bias among contributing studies. | Tables S3, S5, and S6 |
|  | 20b | Present results of all statistical syntheses conducted. If meta-analysis was done, present for each the summary estimate and its precision (e.g. confidence/credible interval) and measures of statistical heterogeneity. If comparing groups, describe the direction of the effect. | p. 7-9; Tables 1 and 3 |
|  | 20c | Present results of all investigations of possible causes of heterogeneity among study results. | p. 8-10; Tables 2 and 4; Table S4 |
|  | 20d | Present results of all sensitivity analyses conducted to assess the robustness of the synthesized results. | p. 8; Table S4 |
| Reporting biases | 21 | Present assessments of risk of bias due to missing results (arising from reporting biases) for each synthesis assessed. | NA |
| Certainty of evidence | 22 | Present assessments of certainty (or confidence) in the body of evidence for each outcome assessed. | NA |
| **DISCUSSION** | | |  |
| Discussion | 23a | Provide a general interpretation of the results in the context of other evidence. | p. 10-11 |
|  | 23b | Discuss any limitations of the evidence included in the review. | p. 12 |
|  | 23c | Discuss any limitations of the review processes used. | p. 12 |
|  | 23d | Discuss implications of the results for practice, policy, and future research. | p. 112 |
| **OTHER INFORMATION** | | |  |
| Registration and protocol | 24a | Provide registration information for the review, including register name and registration number, or state that the review was not registered. | NA |
|  | 24b | Indicate where the review protocol can be accessed, or state that a protocol was not prepared. | NA |
|  | 24c | Describe and explain any amendments to information provided at registration or in the protocol. | NA |
| Support | 25 | Describe sources of financial or non-financial support for the review, and the role of the funders or sponsors in the review. | p. 13 |
| Competing interests | 26 | Declare any competing interests of review authors. | p. 13 |
| Availability of data, code and other materials | 27 | Report which of the following are publicly available and where they can be found: template data collection forms; data extracted from included studies; data used for all analyses; analytic code; any other materials used in the review. | p. 13 |

Abbreviations: NA, not applicable; p, page(s).

# **Table S2.** Data sources and search criteria for systematically reviewing HSV-1 epidemiology in Australia, New Zealand, and Pacific Islands.

| **PubMed (last searched 6 December, 2021):** |
| --- |
| (Simplexvirus[MeSH] OR Herpes Simplex[MeSH] OR herpes genitalis[MESH] OR genital herpes[Text] OR Herpes Genitalis[Text] OR Stomatitis Herpetic[Text] OR Herpes Labialis[Text] OR Human herpes virus[Text] OR Herpes Hominis[Text] OR Herpes virus[Text] OR HSV type-1[Text] OR HSV type 1[Text] OR HSV1[Text] OR HSV-1[Text] OR HSV 1[Text] OR Herpes simplex virus type 1[Text] OR Herpes simplex virus type-1[Text] OR herpes simplex virus 1[Text] OR herpes simplex virus-1[Text] OR herpes simplex type 1[Text] OR herpes simplex type-1[Text] OR herpes simplex 1[Text] OR herpes simplex-1[Text] OR Herpesvirus type 1[Text] OR Herpesvirus type-1[Text] OR Herpesvirus 1[Text] OR Herpesvirus-1[Text] OR Herpes virus type 1[Text] OR Herpes virus type-1[Text] OR Herpes virus 1[Text] OR Herpes virus-1[Text] OR HSV type-2[Text] OR HSV type 2[Text] OR HSV2[Text] OR HSV-2[Text] OR HSV [Text] OR Herpes simplex virus type 2[Text] OR Herpes simplex virus type-2[Text] OR herpes simplex virus 2[Text] OR herpes simplex virus-2[Text] OR herpes simplex type 2[Text] OR herpes simplex type-2[Text] OR herpes simplex 2[Text] OR herpes simplex-2[Text] OR Herpesvirus type 2[Text] OR Herpesvirus type-2[Text] OR Herpesvirus 2[Text] OR Herpesvirus-2[Text] OR Herpes virus type 2[Text] OR Herpes virus type-2[Text] OR Herpes virus-2[Text]) **AND** (Pacific islands[MeSH] OR Oceania[MeSH] OR Australia[MeSH] OR New Zealand [MeSH] OR Pacific Islands[Text] OR Australia*[Text] OR New Zealand*[Text] OR Fiji*[Text] OR New Caledonia*[Text] OR Solomon Island*[Text] OR Ni-Vanuatu[Text] OR Guam*[Text] OR I-Kiribati[Text] OR Marshall*[Text] OR Micronesia*[Text] OR Nauru*[Text] OR Northern Mariana Island*[Text] OR Palau*[Text] OR American Samoa*[Text] OR Cook Island*[Text] OR French Polynesia*[Text] OR Niue*[Text] OR Pitcairn*[Text] OR Samoa*[Text] OR Tokelau*[Text] OR Tonga*[Text] OR Tuvalu*[Text] OR Wallisian Futun*[Text] OR Vanuatu[Text] OR Kiribati[Text] OR Marshall Islands[Text] OR Micronesia[Text] OR Federated States of Micronesia[Text] OR Wallis and Futuna Islands[Text]) |
| **Embase (last searched 6 December, 2021):** |
| exp Herpes simplex/ or exp Simplexvirus/ or exp herpes simplex virus/ or exp genital herpes/ **OR** (Herpes simplex or Herpes simplex virus or human herpes virus or genital herpes or Herpes Genitalis or herpes labialis or herpetic stomatitis).mp. **OR** (HSV type-1 or HSV type 1 or HSV1 or HSV-1 or Herpes simplex virus type 1 or Herpes simplex virus type-1 or herpes simplex virus 1 or herpes simplex virus-1 or herpes simplex type 1 or herpes simplex type-1 or herpes simplex 1 or herpes simplex-1 or Herpesvirus type 1 or Herpesvirus type-1 or Herpesvirus 1 or Herpesvirus-1 or Herpes virus type 1 or Herpes virus type-1 or Herpes virus 1 or Herpes virus-1).mp. **OR** (HSV type-2 or HSV type 2 or HSV2 or HSV-2 or HSV 2 or Herpes simplex virus type 2 or Herpes simplex virus type-2 or herpes simplex virus 2 or herpes simplex virus-2 or herpes simplex type 2 or herpes simplex type-2 or herpes simplex 2 or herpes simplex-2 or Herpesvirus type 2 or Herpesvirus type-2 or Herpesvirus 2 or Herpesvirus-2 or Herpes virus type 2 or Herpes virus type-2 or Herpes virus 2 or Herpes virus-2).mp. **AND** exp New Zealand/ or exp Australia/ or exp Western Australia/ or exp South Australia/ or exp Fiji/ or exp French Polynesia/ or exp Guam/ or exp Kiribati/ or exp Palau/ or exp Pitcairn/ or exp Samoa/ or exp Tokelau/ or exp Tonga/ or exp Tuvalu/ or exp Melanesia/ or exp Pacific islands/ or exp New Caledonia/ or exp Solomon island/ **OR** (Australia* or Fiji* or Marshall* or Micronesia* or Nauru* or Ni-Vanuatu or Guam* or I-Kiribati or Palau* or new Caledonia* or Niue* or Pitcairn* or Samoa* or Tokelau* or Tonga* or Tuvalu* or Vanuatu or Kiribati or New Zealand* or Northern Mariana Island* or Melanesian or American Samoa* or Cook Island* or French Polynesia* or Solomon island or Marshall Islands* or Federated States of Micronesia*).mp.  **OR** (Territory of the Wallis and Futuna Islands*).mp. |

Abbreviations: HSV-1, herpes simplex virus type 1; HSV-2, herpes simplex virus type.

# **Table S3.** Studies reporting HSV-1 seroprevalence among different populations in Australia and Pacific Island nations.

| **Author, year** | **Year(s) of data collection** | **Country** | **Study site** | **Study design^a^** | **Sampling methodology** | **HSV-1 serological assay** | **Population** | **Sample size** | **HSV-1 seroprevalence (%)** |
| --- | --- | --- | --- | --- | --- | --- | --- | --- | --- |
| **General healthy populations** | | | | | | | | | |
| Bowden, 2005 [2] | 2002-03 | Australia | Community | CS | Conv | ELISA | Students in last two years of secondary school | 637 | 32.5 |
| Brazzale, 2010 [3] | 2007-08 | Australia | Outpatient clinic | CS | Conv | ELISA | Indigenous individuals living in Cape York | 270 | 97.8 |
| Cunningham, 1993 [4] | - | Australia | Outpatient clinic | CS | Conv | WB | HSV-2-negative women attending an antenatal clinic | 196 | 81.6 |
| Cunningham, 2006 [5] | 1999-00 | Australia | Community | CS | SRS | WB | General population in Australia | 1,000 | 76.0 |
| Haddow, 2007 [6] | 1999-00 | Vanuatu | Outpatient clinic | CS | Conv | WB | Pregnant women attending an antenatal clinic | 134 | 100.0 |
| Tideman, 2001 [7] | 1995-98 | Australia | Outpatient clinic | CS | Conv | ELISA | Pregnant women attending an antenatal clinic | 408 | 79.2 |
| **Clinical populations** | | | | | | | | | |
| Bassett, 1994 [8] | 1990-91 | Australia | Outpatient clinic | CS | Conv | WB | Heterosexual men attending an STI clinic | 300 | 9.0 |
| Burkhardt, 2021 [9] | 2001-04 | Australia | Outpatient clinic | CS | Conv | ELISA | Patients with ultra-high risk of psychosis | 96 | 44.8 |
| Cunningham, 1993 [4] | - | Australia | Outpatient clinic | CS | Conv | WB | HSV-2-positive women attending an antenatal clinic | 33 | 69.7 |
| Cunningham, 1993 [4] | - | Australia | Outpatient clinic | CS | Conv | WB | HSV-2-positive patients attending an STI clinic | 43 | 67.4 |
| Cunningham, 1993 [4] | - | Australia | Outpatient clinic | CS | Conv | WB | HSV-2-negative patients attending an STI clinic | 64 | 73.4 |
| Russell, 2001 [10] | 1999-00 | Australia | Outpatient clinic | CS | Conv | ELISA | Homosexual men attending an STI clinic | 300 | 73.3 |
| Smith, 2000 [11] | 1997 | Australia | Outpatient clinic | CS | Conv | EIA | Patients attending an STI clinic at baseline | 180 | 68.3 |
| **Oher populations** | | | | | | | | | |
| Jin, 2006 [12] | 2001-05 | Australia | Community | Cohort | Conv | WB | MSM who are HIV-negative | 1,371 | 75.2 |

^a^The reported study design is the original study design (cross-sectional, cohort, or randomized controlled trial). The included seroprevalence measures are those for the baseline measures at the beginning of the study.

Abbreviations: Conv, convenience; CS, cross-sectional; EIA, enzyme immunoassay; ELISA, enzyme-linked immunosorbent type-specific assay; HIV, human immunodeficiency virus; HSV-1, herpes simplex virus type 1; HSV-2, herpes simplex virus type 2; MSM, men who have sex with men; SRS, stratified random sampling; STI, sexually transmitted infection; WB, western blot.

# **Figure S1.** Forest plots for the pooled mean HSV-1 seroprevalence among different populations in Australia.





*Each line represents an HSV-1 seroprevalence measure in a specific stratum.

Abbreviations: HIV, human immunodeficiency virus; HSV-2, herpes simplex virus type 2; MSM, men who have sex with men; STI, sexually transmitted infection.

# **Table S4**. Univariable and multivariable meta-regression analyses for HSV-1 seroprevalence in Australia^a^ using the year of publication instead of the year of data collection as the time variable.

|  | | | | **Outcome measures** | **Samples** | **Univariable analysis** | | | | **Multivariable analysis^b^** | | | |
| --- | --- | --- | --- | --- | --- | --- | --- | --- | --- | --- | --- | --- | --- |
|  |  |  |  |  |  |  |  |  |  | **Model 3^c^** | | **Model 4^d^** | |
|  | | | | **Total n** | **Total N** | ***RR* (95% CI)** | **p-value** | **LR test p-value** | **Adjusted R^2^ (%)** | ***ARR* (95% CI)** | **p-value** | ***ARR* (95% CI)** | **p-value** |
| **Population Characteristics** | **Age group** | <35 years | | 7 | 1,650 | 1.00 | - | 0.173 | 7.79 | 1.00 | - | 1.00 | - |
|  |  | ≥35 years | | 11 | 1,724 | 1.35 (0.88-2.08) | 0.162 |  |  | 1.38 (0.91-2.09) | 0.123 | 1.13 (0.73-1.75) | 0.559 |
|  |  | Mixed | | 9 | 1,524 | 0.95 (0.61-1.50) | 0.832 |  |  | 3.55 (0.97-13.03) | 0.055 | 3.14 (0.96-10.14) | 0.057 |
|  | **Sex^e^** | Women | | 3 | 637 | 1.00 | - | 0.461 | 0.00 | 1.00 | - | 1.00 | - |
|  |  | Men | | 8 | 1,971 | 0.77 (0.41-1.45) | 0.397 |  |  | 0.65 (0.28-1.50) | 0.292 | 0.68 (0.29-1.56) | 0.337 |
|  |  | Mixed | | 16 | 2,290 | 0.97 (0.54-1.75) | 0.922 |  |  | 1.35 (0.57-3.19) | 0.474 | 1.18 (0.51-2.70) | 0.684 |
|  | **Population type** | Healthy general populations | | 14 | 2,511 | 1.00 | - | 0.135 | 9.11 | 1.00 | - | 1.00 | - |
|  |  | Clinical populations | | 8 | 1,016 | 0.67 (0.45-1.00) | 0.051 |  |  | 0.57 (0.27-1.22) | 0.138 | 0.68 (0.34-1.37) | 0.266 |
|  |  | Other populations^f^ | | 5 | 1,371 | 0.94 (0.59-1.48) | 0.779 |  |  | 2.23 (0.93-5.33) | 0.069 | 1.56 (0.69-3.53) | 0.268 |
| **Study methodology characteristics** | **Assay type** | Western blot | | 17 | 3,187 | 1.00 | - | 0.517 | 0.00 | - | - | - | - |
|  |  | ELISA | | 10 | 1,711 | 1.13 (0.78-1.64) | 0.517 |  |  | - | - | - | - |
|  | **Sample size^g^** | <100 | | 1 | 94 | 1.00 | - | 0.335 | 0.58 | - | - | - | - |
|  |  | ≥100 | | 26 | 4,802 | 1.59 (0.60-4.17) | 0.335 |  |  | - | - | - | - |
|  | **Sampling method** | Probability based | | 6 | 1,000 | 1.00 | - | 0.584 | 0.00 | - | - | - | - |
|  |  | Non-probability based | | 21 | 3,898 | 0.89 (0.58-1.37) | 0.584 |  |  | - | - | - | - |
|  | **Response rate** | ≥80% | | 5 | 1,371 | 1.00 | - | 0.939 | 0.00 | - | - | - | - |
|  |  | <80% | | 7 | 1,637 | 0.91 (0.52-1.60) | 0.734 |  |  | - | - | - | - |
|  |  | Unclear | | 15 | 1,890 | 0.93 (0.57-1.53) | 0.772 |  |  | - | - | - | - |
| **Temporal variables** | **Year of publication category** | | ≤2005 | 10 | 2,161 | 1.00 | - | 0.053 | 12.87 | 1.00 | - | - | - |
|  |  |  | >2005 | 17 | 2,737 | 1.41 (0.99-2.00) | 0.053 |  |  | 2.38 (0.95-5.91) | 0.062 | - | - |
|  | **Year of publication** | | | 27 | 4,898 | 1.02 (0.99-1.05) | 0.205 | 0.205 | 2.56 | - | - | 1.05 (0.99-1.12) | 0.068 |

^a^The only one available study from the Pacific Islands nations (Vanuatu) was excluded from this analysis.

^b^Two multivariable models were conducted, one for year of publication as a categorical variable and one for year of publication as a linear term.

^c^Variance explained by the final multivariable model 3 (adjusted *R^2^*) = 21.05%.

^d^Variance explained by the final multivariable model 4 (adjusted *R^2^*) = 24.74%.

^e^Although the sex variable and the year of publication as a continuous linear variable did not have a statistically significant association with the outcome in the univariable analysis (p-value>0.2), they were included in the multivariable analysis because of epidemiological relevance.

^f^Other populations included only men who have sex with men.

^g^Sample size denotes the sample size of each study population found in the original publication.

Abbreviations: *ARR*, adjusted risk ratio; CI, confidence interval; ELISA, enzyme-linked immunosorbent type-specific assay; HSV-1, herpes simplex virus type 1; *RR*, risk ratio.

# **Table S5.** Studies reporting proportions of HSV-1 detection in clinically diagnosed genital ulcer disease and in laboratory-confirmed genital herpes in Australia and New Zealand.

| **Author, year** | **Year(s) of data collection** | **Country** | **Study site** | **Study design^a^** | **Sampling methodology** | **HSV-1 biological assay** | **Population** | **Sample size** | **Proportion of HSV-1 detection (%)** |
| --- | --- | --- | --- | --- | --- | --- | --- | --- | --- |
| **Patients with clinically diagnosed genital ulcer disease** | | | | | | | | | |
| Birch, 2003 [13] | 1999-01 | Australia | Outpatient clinic | CS | Conv | PCR | Patients attending different clinics with genital lesions | 6,210 | 13.1 |
| Bissessor, 2013 [14] | 2003-11 | Australia | Outpatient clinic | CS | Conv | PCR | MSM with ulcers | 30 | 30.0 |
| Mackay, 2006 [15] | 2002 | Australia | Outpatient clinic | CS | Conv | PCR | Patients with acute or chronic genital lesions | 55 | 1.8 |
| Towns, 2016 [16] | 2009-14 | Australia | Outpatient clinic | CS | Conv | PCR | Men with anogenital lesions | 183 | 1.1 |
| **Patients with laboratory-confirmed genital herpes** | | | | | | | | | |
| Birch, 2003 [13] | 1999-01 | Australia | Outpatient clinic | CS | Conv | PCR | Patients attending different clinics with genital lesions | 2,185 | 37.2 |
| Bissessor, 2013 [14] | 2003-11 | Australia | Outpatient clinic | CS | Conv | PCR | MSM with ulcers | 25 | 36.0 |
| Durukan, 2018 [17] | 2004-17 | Australia | Outpatient clinic | Cohort | Conv | PCR | Patients with anogenital HSV infection | 4,517 | 49.7 |
| Field, 1992 [18] | - | Australia | Outpatient clinic | CS | Conv | Culture | Patients with HSV lesions | 356 | 10.1 |
| Gray, 2008 [19] | 1997-06 | New Zealand | Outpatient clinic | Cohort | Conv | Mixed^b^ | Anogenital specimens from patients | 3,933 | 37.0 |
| Knox, 2011 [20] | 2004-05 | Australia | Outpatient clinic | CS | Conv | PCR | Men with anogenital herpes | 155 | 33.6 |
| Knox, 2011 [20] | 2004-05 | Australia | Outpatient clinic | CS | Conv | PCR | Women with anogenital herpes | 475 | 47.6 |
| Ryder, 2009 [21] | 1992-06 | Australia | Outpatient clinic | Cohort | Conv | Mixed^c^ | Patients with first episode anogenital herpes | 1,845 | 35.0 |
| Tran, 2004 [22] | 1980-03 | Australia | Outpatient clinic | Cohort | Conv | Mixed^d^ | Men with genital lesions | 10,426 | 16.4 |
| Tran, 2004 [22] | 1980-03 | Australia | Outpatient clinic | Cohort | Conv | Mixed^d^ | Women with genital lesions | 14,946 | 31.8 |

^a^The reported study design is the original study design (cross-sectional, cohort, or randomized controlled trial). The included measures for the proportion of HSV-1 detection are those for the baseline measures at the beginning of the study.

^b^Mixed^:^ Monoclonal direct fluorescent antibody assay was used, then from late 2002, a PCR assay was used.

^c^Mixed: Cell culture was used until August 2004, after which a validated in-house PCR assay was used.

^d^Mixed: From 1980-1999 a neutralization assay incorporating serotype-specific antibodies was used, then from late 1999, a PCR assay was used.

Abbreviations: Conv, convenience; CS, cross sectional; HSV, herpes simplex virus; HSV-1, herpes simplex virus type 1; MSM, men who have sex with men; PCR, polymerase chain reaction.

# **Figure S2.** Forest plots for the pooled mean proportions of HSV-1 detection in clinically diagnosed genital ulcer disease and in laboratory-confirmed genital herpes in Australia and New Zealand.

## Patients with clinically diagnosed genital ulcer disease.





## Patients with laboratory-confirmed genital herpes.





*Each line represents a proportion measure of HSV-1 detection in genital herpes in a specific stratum.

Abbreviations: HSV, herpes simplex virus; MSM, men who have sex with men.

# **Table S6.** Summary of the precision assessment and risk of bias assessment for the studies reporting HSV-1 seroprevalence in Australia and Pacific Island nations.

| **Quality assessment** | **HSV-1 seroprevalence measures** | |
| --- | --- | --- |
|  | **Number of studies** | **%** |
| **Precision of seroprevalence measures^a^** | | |
| Low precision | 0 | 0.0 |
| High precision | 14 | 100.0 |
| **Risk of bias quality domain^b^** | | |
| **Sampling methodology** | | |
| Low risk of bias | 1 | 7.1 |
| High risk of bias | 13 | 92.9 |
| **Response rate** | | |
| Low risk of bias | 1 | 7.1 |
| High risk of bias | 2 | 14.3 |
| Unclear risk of bias | 11 | 78.6 |
| **Summary of the risk of bias assessment** | | |
| **Low risk of bias** |  |  |
| In at least one quality domain | 2 | 14.3 |
| In both quality domains | 0 | 0.0 |
| **High risk of bias** |  |  |
| In at least one quality domain | 14 | 100.0 |
| In both quality domains | 1 | 7.1 |
| **Seroprevalence studies where risk of bias assessment was possible** | **14** | **100.0** |

^a^Precision was assessed based on the overall sample size (not each stratum subsample size) of the study as reported in the publication.

^b^Risk of bias was assessed based on the overall sample size (not each stratum subsample size) of the study as reported in the publication.

Abbreviation: HSV-1, herpes simplex virus type 1.

# **References**

(1) **Page MJ, et al.** The PRISMA 2020 statement: an updated guideline for reporting systematic reviews. *BMJ* 2021; **372**: n71.

(2) **Bowden FJ, et al.** Sexually transmitted infections, blood-borne viruses and risk behaviour in an Australian senior high school population--the SHLiRP study. *Sexual Health* 2005; **2**(4): 229-236.

(3) **Brazzale AG, et al.** Seroprevalence of herpes simplex virus type 1 and type 2 among the Indigenous population of Cape York, Far North Queensland, Australia. *Sexual Health* 2010; **7**(4): 453-459.

(4) **Cunningham AL, et al.** Herpes simplex virus type 2 antibody in patients attending antenatal or STD clinics. *The Medical Journal of Australia* 1993; **158**(8): 525-528.

(5) **Cunningham AL, et al.** Prevalence of infection with herpes simplex virus types 1 and 2 in Australia: a nationwide population based survey. *Sexually Transmitted Infections* 2006; **82**(2): 164-168.

(6) **Haddow LJ, et al.** Herpes simplex virus type 2 (HSV-2) infection in women attending an antenatal clinic in the South Pacific island nation of Vanuatu. *Sexually Transmitted Diseases* 2007; **34**(5): 258-261.

(7) **Tideman RL, et al.** Sexual and demographic risk factors for herpes simplex type 1 and 2 in women attending an antenatal clinic. *Sexually Transmitted Infections* 2001; **77(6)**: 413-415.

(8) **Bassett I, et al.** Herpes simplex virus type 2 infection of heterosexual men attending a sexual health centre. *The Medical Journal of Australia* 1994; **160**(11): 697-700.

(9) **Burkhardt E, et al.** Toxoplasma gondii, Herpesviridae and long-term risk of transition to first-episode psychosis in an ultra high-risk sample. *Schizophrenia Research* 2021; **233**: 24-30.

(10) **Russell DB, et al.** Seroprevalence of herpes simplex virus types 1 and 2 in HIV-infected and uninfected homosexual men in a primary care setting. *Journal of Clinical Virology* 2001; **22**(3): 305-313.

(11) **Smith A, et al.** Psychosocial impact of type-specific herpes simplex serological testing on asymptomatic sexual health clinic attendees. *International Journal of STD & AIDS* 2000; **11**(1): 15-20.

(12) **Jin F, et al.** Transmission of herpes simplex virus types 1 and 2 in a prospective cohort of HIV-negative gay men: the health in men study. *The Journal of Infectious Diseases* 2006; **194**(5): 561-570.

(13) **Birch CJ, et al.** Detection of varicella zoster virus in genital specimens using a multiplex polymerase chain reaction. *Sexually Transmitted Infections* 2003; **79**(4): 298-300.

(14) **Bissessor M, et al.** The etiology of infectious proctitis in men who have sex with men differs according to HIV status. *Sexually Transmitted Diseases* 2013; **40**(10): 768-770.

(15) **Mackay IM, et al.** Detection and discrimination of herpes simplex viruses, Haemophilus ducreyi, Treponema pallidum, and Calymmatobacterium (Klebsiella) granulomatis from genital ulcers. *Clinical Infectious Diseases* 2006; **42**(10): 1431-1438.

(16) **Towns JM, et al.** Painful and multiple anogenital lesions are common in men with Treponema pallidum PCR-positive primary syphilis without herpes simplex virus coinfection: a cross-sectional clinic-based study. *Sexually Transmitted Infections* 2016; **92**(2): 110-115.

(17) **Durukan D, et al.** Increasing proportion of herpes simplex virus type 1 among women and men diagnosed with first-episode anogenital herpes: a retrospective observational study over 14 years in Melbourne, Australia. *Sexually Transmitted Infections* 2019; **95**(4): 307-313.

(18) **Field PR, Ho DW, Cunningham AL.** The diagnosis of recent herpes simplex virus type 2 genital infections by the simplex-2 test. *Pathology* 1992; **24**(4): 302-306.

(19) **Gray E, Morgan J, Lindeman J.** Herpes simplex type 1 versus Herpes simplex type 2 in anogenital herpes; a 10 year study from the Waikato region of New Zealand. *New Zealand Medical Journal* 2008; **121**(1271): 43-50.

(20) **Knox J, et al.** Age-specific prevalence of herpes simplex viruses in Melbourne. *Pathology* 2011; **43**(1): 64-66.

(21) **Ryder N, et al.** Increasing role of herpes simplex virus type 1 in first-episode anogenital herpes in heterosexual women and younger men who have sex with men, 1992-2006. *Sexually Transmitted Infections* 2009; **85**(6): 416-419.

(22) **Tran T, et al.** Changing epidemiology of genital herpes simplex virus infection in Melbourne, Australia, between 1980 and 2003. *Sexually Transmitted Infections* 2004; **80**(4): 277-279.
